# Isolation and characterization of a resident tolerant *Saccharomyces cerevisiae* strain from a spent sulfite liquor fermentation plant

**DOI:** 10.1186/2191-0855-2-68

**Published:** 2012-12-13

**Authors:** Violeta Sànchez i Nogué, Maurizio Bettiga, Marie F Gorwa-Grauslund

**Affiliations:** 1 Division of Applied Microbiology, Lund University, P.O. Box 124, SE-221 00, Lund, Sweden; 2 Current address: Industrial Biotechnology, Department of Chemical and Biological Engineering, Chalmers University of Technology, Göteborg, Sweden

**Keywords:** *Saccharomyces cerevisiae*, Spent sulfite liquor fermentation, PCR-fingerprinting, Stress tolerance, Resident yeast

## Abstract

Spent Sulfite Liquor (SSL) from wood pulping facilities is a sugar rich effluent that can be used as feedstock for ethanol production. However, depending on the pulping process conditions, the release of monosaccharides also generates a range of compounds that negatively affect microbial fermentation. In the present study, we investigated whether endogenous yeasts in SSL-based ethanol plant could represent a source of *Saccharomyces cerevisiae* strains with a naturally acquired tolerance towards this inhibitory environment. Two isolation processes were performed, before and after the re-inoculation of the plant with a commercial baker’s yeast strain. The isolates were clustered by DNA fingerprinting and a recurrent *Saccharomyces cerevisiae* strain, different from the inoculated commercial baker’s yeast strain, was isolated. The strain, named TMB3720, flocculated heavily and presented high furaldehyde reductase activity. During fermentation of undiluted SSL, TMB3720 displayed a 4-fold higher ethanol production rate and 1.8-fold higher ethanol yield as compared to the commercial baker’s yeast. Another non-*Saccharomyces cerevisiae* species, identified as the pentose utilizing *Pichia galeiformis*, was also recovered in the last tanks of the process where the hexose to pentose sugar ratio and the inhibitory pressure are expected to be the lowest.

## Introduction

Lignocellulosic biomass, composed of cellulose, hemicellulose and lignin, represents a potential source of fermentable sugars for the production of fuels and bulk chemicals (Wyman and Goodman 1993; Hahn-Hägerdal et al. 2006). In ethanol plants, the heat pretreated biomass is hydrolysed and all sugars are potential substrates for ethanol production (recently reviewed by (Alvira et al. 2010)). In pulp and paper mills, however, the cellulose fraction can be used for the production of pulp, paper, board and cellulose-based products. This requires treating wood biomass with a cooking liquor to obtain the discrete fibres. During the process, the monomeric sugars from the hemicellulose fraction are also released into the cooking liquor during the delignification process (Biermann 1996). When the generated cellulose pulp is removed from the cooking liquor, the resulting by-product stream (e.g. spent sulfite liquor (SSL) when using sulfite-based liquor), that contains the remaining monomeric sugars, can be used for the production of ethanol as a co-product (see e.g. (Borregaard (2012); Domsjö Fabriker AB (2012))). In both types of industrial processes, baker’s yeast *Saccharomyces cerevisiae* is traditionally chosen for ethanol production due to its high tolerance towards high concentrations of sugars and ethanol (Rudolf et al. 2009).

Inhibitory compounds can be released during the pretreatment of lignocellulosic feedstock: at high temperature, dehydration of hexose and pentose sugars generates 5-hydroxymethyl-2-furaldehyde (HMF) and 2-furaldehyde (furfural), respectively. Further degradation of HMF leads to the formation of levulinic and formic acids. In addition, acetic acid can be generated from the deacetylation of the hemicellulose fraction (Dunlop 1948; Ulbricht et al. 1984; Palmqvist and Hahn-Hägerdal 2000). Finally, several phenolic compounds, such as vanillin, guaiacol and coniferyl aldehyde, can be present in lignocellulose hydrolysates as a result of lignin degradation (see e.g. (Larsson et al. 1999b)). All these compounds have the ability, although at different levels, to inhibit microbial activity during ethanol fermentation, thereby resulting in increased lag phase, reduced ethanol production rate and/or reduced final ethanol yield (Boyer et al. 1992; Navarro 1994; Larsson et al. 1999a; Almeida et al. 2007).

Additionally, due to the high cost of sterilization, ethanol production is usually performed under non-sterile conditions and runs in continuous or semi-continuous mode with cell recirculation (Narendranath et al. 1997; de Souza Liberal et al. 2005). This favours the introduction of microbial contaminants that can have a negative impact on yeast performance. For example, during a series of batch and continuous fermentations in a corn fibre-based pilot scale facility, *Lactobacillus* species were recurrently found as the main contaminant (Schell et al. 2007). The arabinose fraction of the feedstock was consumed by the bacteria, increasing proportionally the lactic acid level. As a consequence, yeast cell count and ethanol concentration decreased due to the inhibitory effects on the yeast performance by the presence of this organic acid (Schell et al. 2007). Non-sterile conditions and cell recirculation may also favour the introduction and adaptation of wild type yeast species. Lindén and co-workers isolated a *S. cerevisiae* strain able to ferment simultaneously glucose, mannose and galactose in the presence of acetic acid (Lindén et al. 1992). This strain displayed a higher ethanol yield from SSL than the commercial baker’s yeast (Lindén et al. 1992).

In this work, we report on the isolation and identification of a resident yeast species in a SSL ethanol plant that operates in multistage continuous mode with yeast cell recirculation using a mixture of spruce (*Picea abis*) and pine (*Pinus sylvestris*) hydrolysate. Isolates were analysed using a PCR-fingerprinting method and the dominant and recurrently identified yeast strain was characterised.

## Materials and methods

### Strains

Commercial baker’s yeast (Jästbolaget AB, Rotebro, Sweden), TMB3500 (Almeida et al. 2009) and two yeast strains previously isolated from the same spent sulfite fermentation plant, TL3 and TL10, denoted isolate 3 (TMB3000) and isolate 10 in (Lindén et al. 1992), respectively, were used in the study. Strains were maintained on agar plates containing 6.7 g l^-1^ Yeast Nitrogen Base (YNB) medium and 20 l^-1^ glucose.

Material transfer requests for the isolated strain TMB3720 should be addressed to Domsjö Fabriker AB for the attention of Monika Westerlund (monika.westerlund@domsjo.adityabirla.com).

### Yeast isolation

Yeast strains were isolated from three different tanks in the ethanol fermentation line of a Swedish biorefinery that produces dissolving cellulose as the main product in a sodium-based sulfite cooking liquor process. Samples were plated after serial dilution on YPD solid medium containing 10 g l^-1^ yeast extract, 20 g l^-1^ peptone, 20 g l^-1^ glucose and 20 g l^-1^ agar and incubated at 30°C. At least 30 colonies of different morphology were selected arbitrarily and streaked consecutively at least four times to obtain pure colonies.

### DNA extraction, PCR analysis and molecular identification

Yeast strains were grown in YNB medium containing 6.7 g l^-1^ YNB and supplemented with 20 g l^-1^ glucose. Medium was buffered at pH 5.5 with 50 mM potassium hydrogen phthalate (Hahn-Hägerdal et al. 2005). One loopful of each yeast isolate/strain was added to 5 ml YNB medium, in 50 ml conical tubes, incubated at 30°C in a rotary shake-incubator at 200 rpm and harvested at exponential phase. Chromosomal DNA was extracted with a bead-beater (Biospecs Products, Bartlesville, OK, USA) and phenol/chloroform (Sambrook and Russel 2001).

A TY-elements based method, adapted from (Pearson et al. 1995) was used for DNA-fingerprinting of yeast isolates. PCR primer pairs targeting the TY1 *delta* element and TY3 *sigma* element long terminal repeats were used separately to obtain two single TY element fingerprints (Table 1). A combined TY element fingerprint was also obtained by using a four primer multiplex system. PCR was carried out using the following thermal cycler programme: 94°C 5 min; 30 cycles of 94°C 30 s, 43.5°C 45 s, 72°C 2 min; final extension 72°C 7 min. Fingerprints were performed from biological duplicates. Gel images from yeast fingerprints were recorded under UV light in a digital photo-documenting apparatus (Bio-Rad Laboratories, Hercules, CA, USA). Dendrograms were generated to illustrate the similarity among the different yeast fingerprints and were obtained from the analysis of the three different generated fingerprints for each isolate/strain. The length of the amplicons was estimated by comparison with standard molecular markers (GeneRuler DNA Ladder Mix, Fermentas, St Leon-Rot, Germany). Fingerprints analysis and generation of dendrograms were performed using the photocapture software GelCompar (Applied Maths NV, Saint-Martens-Latem, Belgium). Similarity of the band pattern profile was established using the Pearson coefficient and dendrograms were generated using the UPGMA algorithm. A method technical threshold was set to 90% similarity, above which significant differentiation of the isolates was not possible. Therefore, within the typing parameters used for the present study, isolates with similarity score higher than 90% were considered to be to the same strain. 

**Table 1:** Composite primers used in the study

**Aim**	**Primer**	**Nucleotide sequence (5’ → 3’)**	**Reference**
**Yeast fingerprinting**	TY1 forward	GAATCCCAACAATTATCT	(Pearson et al. 1995)
	TY1 reverse	CAATTGTTGATAAAGGCT	(Pearson et al. 1995)
	TY3 forward	ACGGAATGTTACTTATCTT	(Pearson et al. 1995)
	TY3 reverse	GAATTAATCTGATAAACTGT	(Pearson et al. 1995)
**25S rDNA sequencing**	NL1	GCATATCAATAAGCGGAGGAAAAG	(Kurtzman and Robnett 1997)
	NL4	GGTCCGTGTTTCAAGACGG	(Kurtzman and Robnett 1997)

For each generated dendrogram, at least one isolate from each sub-cluster was arbitrarily chosen for species identification by sequencing the D1/D2 region of 25S rDNA (Valente et al. 1999) within a 600-bp fragment amplified with the primers NL1 and NL4 (Table 1) (Kurtzman and Robnett 1997). PCR was performed using the following thermal cycler programme: 94°C 5 min; 30 cycles of 94°C 30 s, 52°C 45 s, 72°C 2 min; final extension 72°C 7 min. PCR products were purified using E.Z.N.A. Cycle-Pure kit (Omega Bio-Tek, Doraville, GA, USA). DNA sequencing service was purchased from Eurofins MWG Operon (Ebersberg, Germany).

Dream Taq DNA polymerase (Fermentas) and High Fidelity PCR mix (Fermentas) were used for analytical and preparative PCR reactions, respectively. DNA fragments were separated in 0.8% agarose gel at 100V cm^-1^ in 0.5xTBE (Tris-Borate-EDTA) stained with ethidium bromide (Sambrook and Russel 2001).

### SSL fermentation

Softwood spent sulfite liquor (SSL) from a mixture of spruce and pine was kindly provided by Roland Agnemo from Domsjö Fabriker AB (Örnsköldsvik, Sweden). The SSL contained a mixture of hexose and pentose sugars as well as 5.3 g l^-1^ acetic acid, 0.5 g l^-1^ HMF and 0.2 g l^-1^ furfural. Pre-cultures, using one loopful of each *S. cerevisiae* isolate/strain, were propagated overnight in 50 ml conical tubes containing 5 ml YNB medium supplemented with 20 g l^-1^ glucose. Biomass for microaerophilic batch fermentations was obtained by growing yeast cells in 50 ml SSL:YNB medium (50% v/v) supplemented with 5 g l^-1^ ammonium sulfate and adjusted to pH 5.0 with concentrated KOH. The medium was inoculated with the pre-culture at an initial optical density (OD) of 0.3 at 620 nm and cells were incubated at 30°C in a rotary shake-incubator at 200 rpm. When late exponential phase was reached, cells were harvested by centrifugation, washed with 0.9% NaCl solution, and added at a final concentration of 20 g wet weight l^-1^ to 50 ml undiluted SSL. Fermentation was performed in small vials sealed with a rubber stopper, equipped with a needle for carbon dioxide removal. Microaerophilic batch fermentations were performed in undiluted SSL supplemented with 5 g l^-1^ ammonium sulfate and adjusted to pH 5.0 using sodium hydroxide pellets to avoid dilution. Vials were incubated at 30°C in a water bath equipped with a multimagnetic stirring plate (Variomag Telesystem, Thermo Scientific, Waltham, MA, USA) at 140 rpm and microaerophilic conditions were kept by using a layer of mineral oil. Fermentation experiments were performed in two biological duplicates.

### Acetic acid aerobic cultures

*S. cerevisiae* pre-cultures were grown in 50 ml conical tubes. One loopful of each strain was added to 5 ml YNB medium supplemented with 20 g l^-1^ glucose and incubated overnight at 30°C in a rotary shake-incubator at 200 rpm. Pre-cultures were used to inoculate 25 ml YNB medium containing acetic acid (0, 6 or 12 g l^-1^), at an initial OD (620nm) of 0.2. Cells were grown in 250 ml shake flasks, and incubated at 30°C in a rotary shake-incubator at 200 rpm. YNB medium was supplemented with 20 g l^-1^ glucose, 50 mM potassium hydrogen phthalate buffer (Hahn-Hägerdal et al. 2005) and acetic acid. The pH of the resulting medium was adjusted to 5.5 with concentrated KOH and the medium was sterile filtered.

### Metabolites determination

Cells were quickly separated from the SSL by centrifugation; the supernatant was filtered through 0.20 μm membrane filters (Toyo Roshi Kaish, Tokyo, Japan) and stored at −20°C until analysis. Concentrations of glucose, galactose, mannose and xylose were determined by high performance liquid chromatography (HPLC) (Waters, Milford, MA, USA). The compounds were separated with two Rezex RPM Monosaccharide Pb^2+^ polymer-based columns (Phenomenex, Torrence, CA, USA) connected in series and preceded by a Carbo-Pb^2+^ SecurityGuard Cartridge (Phenomenex). Separation was performed at 85°C, with H_2_O at a flow rate of 0.5 ml min^-1^ as mobile phase. Concentrations of glycerol, acetate, ethanol, HMF and furfural were determined by HPLC (Waters) using a HPX-87H resin-based column (Bio-Rad, Hercules, CA, USA) preceded by a Micro-Guard Cation-H guard column (Bio-Rad). Separation was performed at 45°C, with 5mM H_2_SO_4_ at flow rate of 0.6 ml min^-1^ as mobile phase. All compounds were quantified by refractive index detection (Shimadzu, Kyoto, Japan). For each HPLC run, a seven-point calibration curve was made for each compound to calculate concentrations. Each sample was analysed at least in duplicate.

Cell concentrations were determined from absorbance measurements at 620 nm on samples diluted to give an optical density (OD) of less than 0.4 (Spectrophotometer U-1800, Hitachi, Berkshire, UK).

### Enzymatic activity measurements

Overnight grown cells in YNB medium with 20 g l^-1^ glucose were used to inoculate, at initial OD (620nm) of 0.2, 500 ml shake flasks containing 50 ml YNB medium with 20g l^-1^ glucose. Strains were grown aerobically at 30°C in a rotary shake-incubator at 200 rpm and harvested in exponential growth phase. Cultivations were performed in biological duplicates. Crude cell extract was prepared with Y-PER reagent following the recommendations of the supplier (Thermo Scientific, Rockford, IL, USA). The protein concentration was determined using Albumin Standard (Thermo Scientific) and Coomassie Protein Assay Reagent (Thermo Scientific). Specific activity was measured as previously reported by Wahlbom *et al.* (Wahlbom and Hahn-Hägerdal 2002). Briefly, cell free extract was diluted in 1 ml of 100 mM phosphate buffer (pH 7.0) and NADH or NADPH was added to a final concentration of 200 μM. The reaction was started by addition of HMF or furfural to a concentration of 10 mM. Reduction of these compounds was followed at 30°C by monitoring the oxidation of NAD(P)H as the change in absorbance at 340 nm (Ultrospec 2100 pro spectrophotometer, Amersham Biosciences, Uppsala, Sweden).

## Results

### Isolation and strain typing

A first yeast isolation was carried out during March 2008 and a second isolation was performed eight months later, after the SSL plant had been regularly re-inoculated with the local commercial baker’s yeast (BY). In both isolations, samples were taken from three different in-series tanks (number 1, 3 and 4). Each sample was plated in serial dilution on YPD plates. Aerobic cultivation on solid rich medium was chosen in order to select *S. cerevisiae* strains as well as any other type of yeast contaminants. In the first isolation, thirteen colonies were arbitrarily selected from the different tanks. In the second isolation, twelve colonies from each tank were selected, also arbitrarily. Each isolated clone was named using a three number code where the first number denoted the isolation number (I); the second number, the tank from where it was isolated (T) and the third, the clone number (C): #I.T.C. DNA fingerprinting of all yeast isolates was then performed by PCR using primers homologous to regions situated in *S. cerevisiae* TY elements (Table 1) (Pearson et al. 1995). As a reference, BY strain that was used to regularly inoculate the SSL plant was included. The analysis also included previously SSL-isolated yeast strains TL3 and TL10 (Lindén et al. 1992).

**Figure 1:**
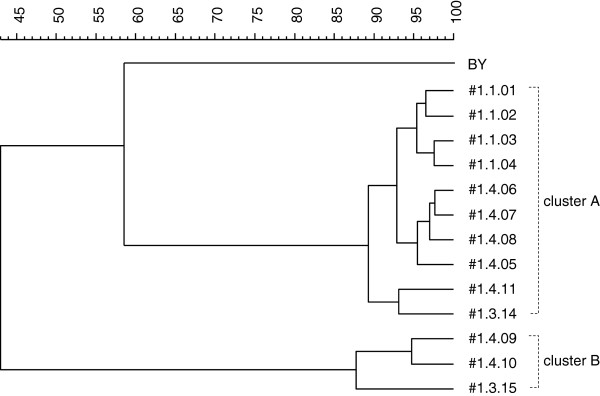
Dendrogram of isolates from the first isolation process and commercial baker’s yeast (BY).

From the dendrogram that contained the reference strain BY and all isolates from the first isolation process, BY clustered in a distinctly separate clade from all isolates (Figure 1). Two additional clearly separated clusters (A and B), having less than 50% similarity, were obtained. Cluster A contained only *S. cerevisiae* strains, originating from all tanks. In contrast, the three isolates from cluster B (#1.3.15, #1.4.09 and #1.4.10) originating from tanks number 3 and 4, were identified as *Pichia* species. In cluster A, all isolates were less than 60% similar to BY and had high internal similarity (over 85%). Within cluster A, three isolates (#1.1.03, #1.4.06 and #1.3.14) which belonged to three highly similar (> 85%) sub-clusters were arbitrarily selected for further characterization. 

**Figure 2:**
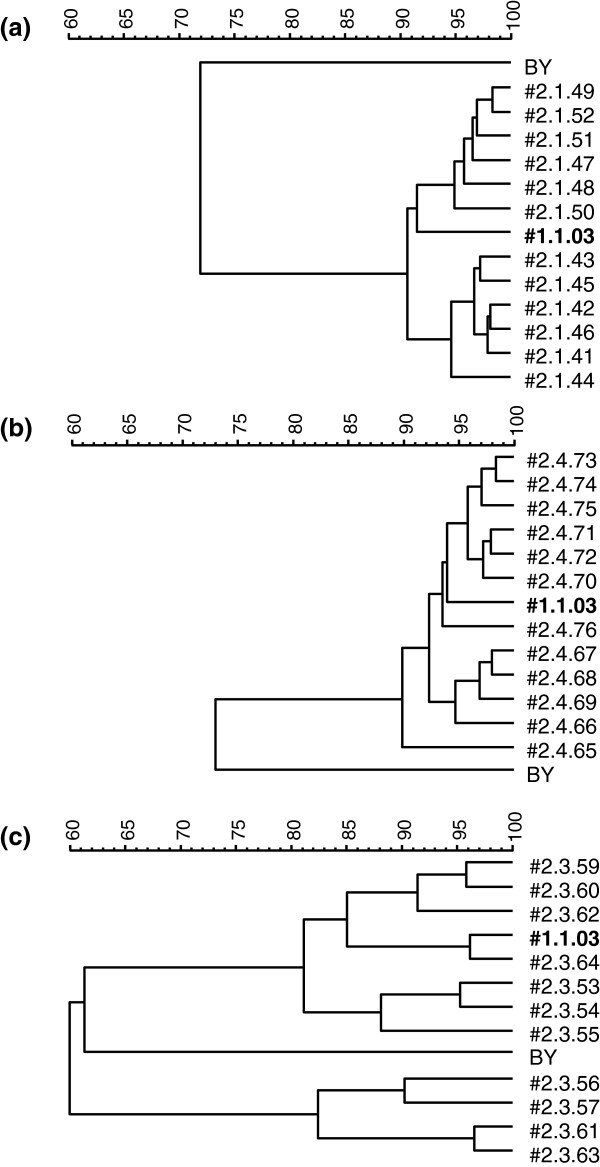
**Dendrogram containing isolates from the second isolation process, isolate #1.1.03 from the first isolation process and commercial baker’s yeast (BY).** (**a**) Tank 1; (**b**) Tank 4 and (**c**) Tank 3.

The process was repeated for samples from the second isolation process and the isolates were also compared to isolate #1.1.03 originating from the first isolation process. Since a higher number of isolates were evaluated, isolates from each tank were treated separately and one dendrogram was generated per tank (Figure 2). Also in this case, very similar profiles were obtained and, consistently with the results obtained for the first isolation process, low similarity was obtained between the isolates and BY (ca. 72%). Isolates from tank 1 had a similarity of at least 90% and they clustered together with isolate #1.1.03, (Figure 2a). Therefore only one isolate (#2.1.48) was chosen for further characterisation. The same pattern was observed for the isolates from tank 4 (Figure 2b). In this case, isolate #2.4.69 was selected for further characterization. In contrast, isolates from tank 3 were placed into two clearly separated clusters with 60% similarity only (Figure 2c). The four isolates belonging to one of the cluster (#2.3.56, #2.3.57, #2.3.61 and #2.3.63) were consistently identified as the yeast *Pichia* sp. In the other cluster, all isolates belonged to a *S. cerevisiae* group that had less than 62% similarity with BY (Figure 2c). One isolate (isolate #2.3.54) was selected to be further characterised. 

**Figure 3:**
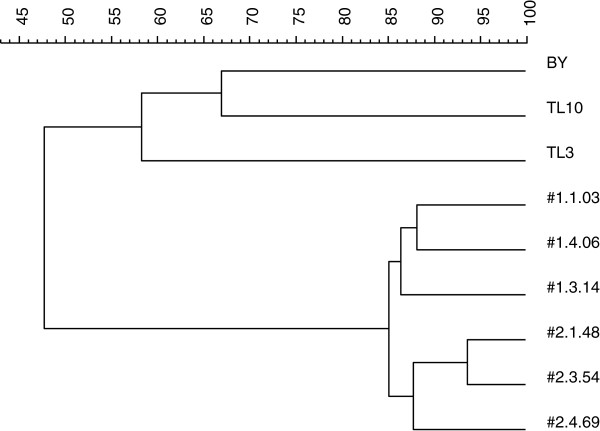
**Dendrogram of isolates from the first and the second isolation processes and controls used in this study.** (Commercial baker’s yeast (BY) and two strains isolated previously from the same plant, TL3 and TL10).

All selected isolates were finally compared with the two previously isolated strains TL3 and TL10 on the same site in 1992 (Lindén et al. 1992) (Figure 3). The new isolates, which all belonged to the same cluster with similarity higher than 80%, appeared to be less than 60% similar to TL3 and TL10. 

### SSL fermentation

The selected isolates (#1.1.03, #1.3.14, #1.4.06, #2.1.48, #2.3.54 and #2.4.69) were tested for anaerobic ethanol production from undiluted softwood SSL and their fermentation performance was compared to BY. Strain TMB3500, previously shown to be tolerant towards several non-detoxified lignocellulosic hydrolysates (Almeida et al. 2009), was also included in the comparison. Biomass for inoculation was obtained by pre-growing the cells aerobically in YNB:SSL medium (50% v/v). Cells were harvested at late exponential phase and used to inoculate 50 ml softwood SSL at pH 5 with 20 g wet weight l^-1^.

**Figure 4:**
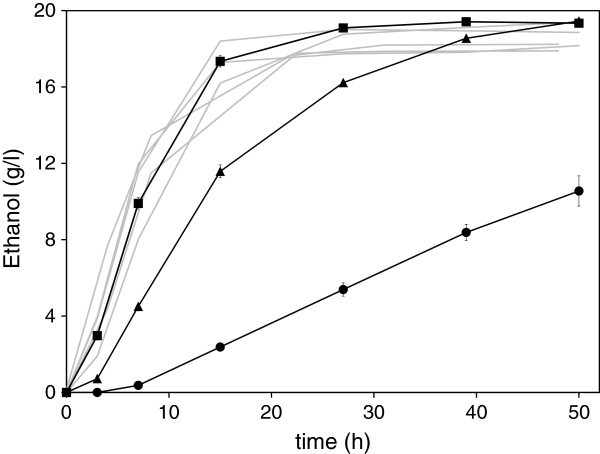
**Anaerobic ethanol production from undiluted SSL.** TMB3720 (squares), BY (circles) and TMB3500 (triangles). Each grey is a representative profile (from 2 or more replicates) of the ethanol production for the tested isolates from the first (#1.3.14, #1.4.06), and the second isolation process (#2.1.48, #2.3.54 and #2.4.69).

Sugar consumption and ethanol production profiles were highly comparable for all selected isolates from both isolation processes (Figure 4). Also, for all tested isolates, no lag phase was observed and the maximum ethanol concentration was very similar (18.5 ± 0.6 g l^-1^, depending on the isolate). These data, in addition to the high similarity of the DNA fingerprints (85%) confirmed that the different isolates were originating from the same strain. Therefore, isolate #1.1.03 was chosen as representative for the comparison with BY and TMB3500 and was named TMB3720. 

TMB3720 and TMB3500 were clearly more efficient ethanol producers than BY, that displayed a lag phase of approximately 3 hours, a low maximum specific ethanol production rate and produced only half of the ethanol concentration (10.5 g l^-1^) after 50 h (Figure 4) (Table 2). TMB3720 and TMB3500 displayed the same ethanol yield of 0.37 g ethanol g hexoses^-1^ (Table 2). However, the maximum specific ethanol production rate was significantly higher for TMB3720, 0.08 g ethanol l^-1^ g wet cells^-1^ h^-1^, compared to 0.05 g ethanol l^-1^ g wet cells^-1^ h^-1^ achieved by TMB3500, which could be explained by the fact that the highest concentration of 19.5 g l^-1^ ethanol was reached only after 50 hours of batch fermentation as compared to 27 hours for TMB3720 (Figure 4). 

**Table 2:** Specific ethanol production rate and yield in anaerobic cultivation of SSL

**Strain**	**Specific Ethanol production rate**	**Ethanol yield**
	**(g g wet cells**^**-1**^**l**^**-1**^**h**^**-1**^**)**	**(g ethanol g hexoses**^**-1**^**)**
TMB3720	0.08 ± 0.02	0.37 ± 0.01
BY	0.02 ± 0.00	0.21 ± 0.02
TMB3500	0.05 ± 0.00	0.38 ± 0.00

### Growth and acetic acid tolerance

Acetic acid released during the hydrolysis of lignocellulosic biomass is known to inhibit yeast performance (Palmqvist and Hahn-Hägerdal 2000). Since the SSL contained more than 5 g l^-1^ of acetic acid, the sensitivity towards this weak acid was also evaluated by comparing growth in the absence and presence of acetic acid. The aerobic growth rate of TMB3720, BY and TMB3500 was determined in YNB medium at pH 5.0 (i.e. the plant operation pH) using glucose as carbon source and containing 0, 6 or 12 g l^-1^ acetic acid. Cells were inoculated at initial OD of 0.2 and growth was followed over time. In the control conditions (i.e., without acetic acid), TMB3720 displayed a maximum specific growth rate of 0.26 ± 0.01 h^-1^, which was significantly lower than TMB3500 and BY (0.45± 0.00 h^-1^ and 0.44± 0.00 h^-1^, respectively) (Table 3). The maximum specific growth rate of TMB3720 was not significantly affected (P < 0.05) by the presence of acetic acid. Also the specific growth rate of TMB3500, was only significantly lower (P < 0.05) when the higher concentration of acetic acid was tested (Table 3). For BY, however, a reduction of 6% and 9% of the specific growth rate was observed in the presence of 6 and 12 g·l^-1^ acetic acid, respectively. In all tested conditions, TMB3720, displayed a high flocculation capability.

**Table 3:** Specific growth rate (h^-1^) for strains growing aerobically on glucose mineral medium with and without acetic acid

**Strain**	**without acetic acid**	**with acetic acid**	
		**6 g l**^**-1**^	**12 g l**^**-1**^
TMB3720	0.26 ± 0.01	0.25 ± 0.00	0.25 ± 0.00
BY	0.44 ± 0.00	0.41 ± 0.00	0.40 ± 0.00
TMB3500	0.45 ± 0.00	0.45 ± 0.01	0.44 ± 0.00

### Furaldehyde reduction

**Figure 5:**
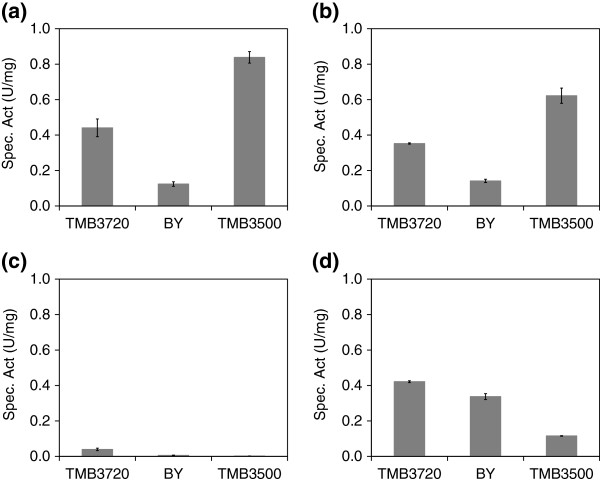
***In vitro* reductase activity.** HMF (left column) and furfural (right column) reduction activity measured in crude cell extracts from cells of TMB3720, BY and TMB3500 using NADPH (top row) or NADH (bottom row) as cofactor.

Each *S. cerevisiae* strain has a given innate tolerance towards furaldehydes such as HMF and furfural, which enable it to reduce them to their less inhibitory alcohols (Villa et al. 1992; Taherzadeh et al. 2000; Liu et al. 2004). The reduction capability of these compounds has been related to the overall fermentation performance in lignocellulosic hydrolysates (Liu et al. 2004; Nilsson et al. 2005; Almeida et al. [Bibr B4][Bibr B5]; Modig et al. 2008). Therefore, furaldehyde reduction was tested by measuring the specific NADH and NADPH-dependent HMF and furfural reductase activity from YNB medium grown cells for strains TMB3720, BY and TMB3500 (Figure 5). TMB3500 displayed the highest NADPH-dependent reductase activity (Figure 5a and 5b), being 1.9-fold and 1.8-fold higher for HMF and furfural, respectively, than for TMB3720. TMB3720 showed the second highest specific activity, with NADPH-dependent HMF reduction that was 3.6-fold higher than for BY. When furfural was used as substrate, TMB3720 also displayed 2.5-fold higher NADPH-dependent reductase activity than BY. 

When NADH-dependent reductase activity was tested, TMB3500 displayed the lowest activity among all the tested strains, whereas TMB3720 showed the highest activity (Figure 5c and 5d). BY and TMB3500 could only reduce furfural, whereas TMB3720 also displayed some NADH-dependent HMF reductase activity (Figure 5c and 5d). This unusual co-factor specificity with HMF as a substrate has only been previously reported for one *S. cerevisiae* strain (TL3/TMB3000) (Nilsson et al. 2005), which was shown, in this study, not to belong to the same strain cluster (Figure 3).

## Discussion

PCR-fingerprinting methods have previously been used to follow the population dynamics in sugar cane-based distilleries over a fermentation season (de Souza et al. 2005; Silva-Filho et al. [Bibr B38][Bibr B39]; Basílio et al. 2008) and during wine fermentation (Xufre et al. 2011). In the present study, a similar method was applied to demonstrate that a resident *S. cerevisiae* strain, different from the inoculum strain, can dominate and repeatedly take over the fermentation process in a multistage continuous SSL fermentation plant. Whereas sugar cane juice and molasses, obtained from the processing of sugar cane, contain already considerable amounts of readily fermentable sugars (Basso et al. 2011), lignocellulosic-based feedstock requires the hydrolysis of polysaccharides to obtain fermentable sugars. As a consequence, inhibitory compounds are also released during the pretreatment step (Almeida et al. 2007), which implies that the obtained strain may differ considerably from previously isolated strains from sugar cane distilleries (Silva-Filho et al. 2005a; Basso et al. 2008). In the two isolation processes that were carried out before and after a regular plant re-inoculation with commercial baker’s yeast (BY), BY was never identified. Instead, all isolates displayed very similar molecular and physiological profiles that were distinct from BY. The identified contaminant yeast strain, named TMB3720, clearly fermented better undiluted softwood SSL than BY, with 4-fold higher maximum specific ethanol productivity and 1.8-fold higher ethanol yield. It also displayed similar ethanol yield and 1.6-fold higher maximum specific ethanol productivity than the previously reported tolerant industrial strain TMB3500 (Almeida et al. 2009).

TMB3720 clustered in a different clade that the strains isolated from the same SSL ethanol plant in the early 90’s (Lindén et al. 1992). The two isolation processes are separated by more than 15 years and by the implementation of four stainless steel fermentation tanks in the SSL plant, which indicates that the modifications in the yeast population result from differences in environmental conditions as well as the possible input of novel wild yeasts species from the raw material (Silva-Filho et al. 2005b). Chronical contamination episodes are actually expected in large scale facilities as bioethanol fermentations are not designed to be carried out under sterile conditions (Skinner and Leathers 2004). However such episodes are often associated with negative fermentation performances. Notably, contamination by lactic acid bacteria is regarded as one of the major problems in ethanol production facilities (Hynes et al. 1997; Narendranath et al. 1997). A reduction up to 7.6% of ethanol concentration was, for example, demonstrated when 10^9^ CFU of lactic acid bacteria/ml were added to wheat mash, as a result of lactic acid production and possible competition with yeast for essential growth factors (Narendranath et al.^ 1997^). Also, an economically relevant decrease of ethanol yield was observed when the level of the yeast *Dekkera bruxellensis,* which was consistently identified as the main contaminant in bioethanol distilleries in Brazil, increased up to almost 50% of the yeast population (de Souza Liberal et al. 2007). In some cases however, microbial contamination did not give detrimental effects. For example, a stable ethanol-producing consortium consisting of *D. bruxellensis* and *Lactobacillus vini* showed the same efficiency and productivity as the inoculated commercial baker’s yeast in a wheat starch-based alcohol production process (Passoth et al. 2007).

In the present study, the contaminating strain, TMB3720, showed even clearly superior traits than the inoculated commercial BY strain in fermenting undiluted softwood SSL, which indicates that the lack of sterility can also be an advantage for the selection of better performing wild yeast strains for SSL fermentation processes.

Acetic acid tolerance and furaldehyde reduction capacity may explain why TMB3720 strain became the resident yeast in the SSL-based fermentation plant. At the plant operation pH, pH 5, the maximum specific growth rate of TMB3720 was not affected by the presence of acetic acid whereas a reduction of 6% was observed for BY. At this pH, 42% of the total acetic acid (6 g l^-1^) remains in undissociated form which diffuses through the cell membrane. Once inside the cell, acetic acid dissociates, thereby releasing protons and acidifying the cytosol. To be able to maintain the intracellular pH level, the excess of protons have to be transported out of the cell using ATP, therefore additional ATP needs to be synthesized. If the ATP production rate becomes limiting, less ATP is available for biomass formation (Pampulha and Loureiro-Dias 2000). Therefore, in the presence of acetic acid, TMB3720 might be able to cope with the extra ATP demand without reducing the biosynthetic capability to produce biomass. Also, TMB3720 showed higher *in vitro* reductase activity for furfural and HMF as compared to BY. The lower reductase capacity of BY together with the fact that it could not be found during the second isolation process, indicates that this strain was not able to cope with the inhibitory conditions of the fermentation plant. Moreover, since yeast is being recirculated, the cell lysis of BY could serve as a source of fresh nutrients for TMB3720.

During the cell propagation step for SSL fermentation and also in the evaluation of the acetic acid tolerance, TMB3720 heavy flocculated when grown in liquid media. A formation of flocs with the subsequent rapid sedimentation from the medium was observed regardless of the type of medium used (defined or rich). The same process of aggregation took place when the cells were grown in the presence of softwood SSL. In parallel, TMB3720 had a significantly lower specific growth rate (0.26 ± 0.01 h^-1^) compared to two other tested industrial strains (0.44 ± 0.00 h^-1^ and 0.45 ± 0.00 h^-1^ for BY and TMB3500 respectively). This fact could be linked to mass transfer limitations inside the floc due its high flocculation capability. However, the variability in the size flocs, together with the fact that they could not be dispersed completely by the use of EDTA, might also lead to an underestimation of the maximum specific growth rate. Flocculation has been suggested to be a social behaviour and to act as a mechanism of survival under nutrient limitation or high ethanol concentrations (Soares 2010). Yeast flocculation depends on the expression of specific flocculation genes such as *FLO1*, *FLO5*, *FLO8* and *FLO11* (Verstrepen et al. 2003). When comparing the non-flocculent S288C *S. cerevisiae* strain and its *FLO1*-overexpressing variant under ethanol stress, the number of the cell survival of the flocculent strain was two-fold greater compared to the wild type, and it was suggested that cells enclosed within the floc would induce changes at physiological level that would promote stress resistance (Smukalla et al. 2008). Therefore, flocculation may also partly explain why TMB3720 predominates under the harsh conditions of the SSL plant. On the one hand, the external part of the floc might act as a protectant that also detoxify compounds for the inside cells. On the other hand, the possible cell lysis could be a provision of nutrients for the metabolic active cells present inside the floc.

In the course of the study, *Pichia galeiformis* yeast contaminant was also isolated in the last fermentation tanks. Xylose-utilising *Pichia* species have previously been identified in the same SSL plant (Lindén et al. 1992). Their presence is most probably connected to the fact that *S. cerevisiae* cannot naturally consume xylose, which would leave this sugar fraction available for xylose-utilising contaminant species at any point of the process line. Also, it is known that another *Pichia* species, *Pichia (Scheffersomyces) stipitis*, is inhibited by compounds present in lignocellulosic biomass (Lohmeier-Vogel et al. 1998). Therefore, we can speculate that the detoxification process performed by *S. cerevisiae* in the fermentation line would reduce the toxicity of the SSL and allow less tolerant contaminant species to be present only at the end of the process line. *Pichia* isolates found at the end of the fermentation line would, however, not be tolerant enough to be a contaminant able to take over the entire fermentation line. The presence of inhibitory compounds, and notably sulfur-derivate compounds (du Toit et al. 2005) may also explain why *Dekkera* species were not found in any of the isolation processes, although it has been identified as a major contaminant species in sugar cane juice distilleries (de Souza Liberal et al. 2007; Basílio et al. 2008; Basso et al. 2008) and is also believed to be the cause of reported stuck fermentations in corn mash-based fermentation processes (Abbott et al. 2005).

In conclusion, TMB3720 is an adapted high-productive and robust *S. cerevisiae* strain that can be employed as regular inoculum in SSL plants. More generally, the isolation and characterization of tolerant high ethanol producing resident yeasts from lignocellulosic biomass plants will increase the knowledge on tolerance to highly inhibitory compounds as well as generate background strains for further improvement by targeted and/or evolutionary engineering.

## Competing interests

The authors declare that they have no competing interests.
